# A genome-wide screening for DNA repair genes: much more players than hitherto known

**DOI:** 10.1038/s41392-020-00314-4

**Published:** 2020-09-15

**Authors:** Bernd Kaina

**Affiliations:** Institute of Toxicology, University Medical Center, Obere Zahlbacher Str. 67, Mainz, Germany

**Keywords:** Genomic instability, Toxicology

**Fig. 1:**
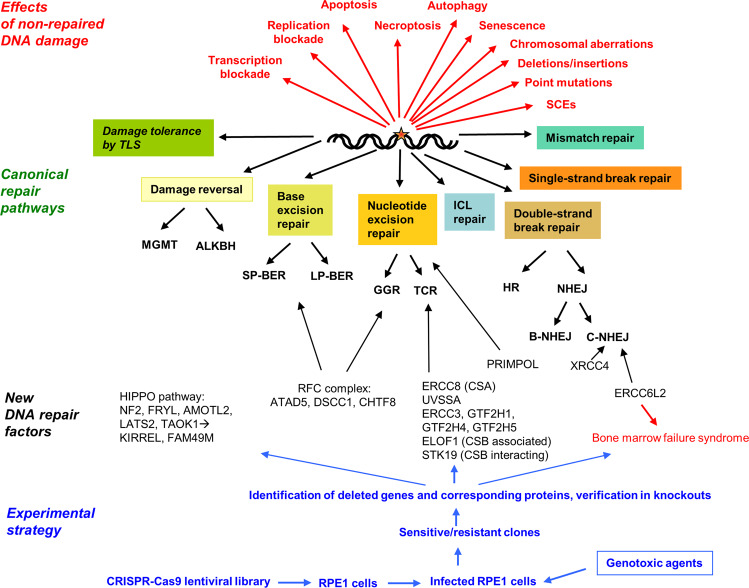
Schematic representation of DNA repair pathways, consequences of non-repaired DNA lesions, the experimental strategy used by the authors and several of the new genes identified. MGMT O^6^-methylguanine-DNA methyltransferase; ALKBH2 alkB homolog protein 2; SP-BER short-patch base excision repair; LP-BER long-patch base excision repair; GGR global genomic repair; TCR transcription-coupled repair; ICL interstrand cross-link; SCEs sister-chromatid exchanges; B-NHEJ backup (alt) nonhomologous end-joining; TLS translesion synthesis

Since the discovery of the structure of DNA and the statement by Francis Crick that DNA is “…so precious that probably many distinct repair mechanisms would exist”,^1^ a plethora of DNA alterations induced by exogenous and endogenous genotoxins has been identified, together with complex cellular processes that sense the damage, remove it from the macromolecule or tolerate it during replication. Kinase-driven pathways signal downstream to complex networks, which regulate the balance between survival and death, and are collectively referred to as the DNA damage response (DDR). The classical DNA repair and DDR pathways have been elucidated in detail and a tight entanglement has been uncovered between ATM-ATR-DNA-PK driven pathways, chromatin remodeling, damage recruiting factors, and mechanisms removing or tolerating DNA damage in a simple (damage reversal) or more complex way (Fig. 1).

Many of the repair and DDR genes were identified by harnessing genotoxin-hypersensitive syndromes or genotoxin-hypersensitive mutant cell lines. This classical approach, however, has limitations. The application of new technologies has given the field new impetus. Thus, the genome-wide induction of genetic changes by means of the CRISPR-Cas9 technology, combined with drug screening, has enabled researchers to identify new gene-drug interaction networks. In a recent paper published in *Cell* by Olivieri et al. this strategy was applied and the authors succeeded in uncovering a number of new DNA repair factors and gained insights into new mechanisms of genotoxic agents.^2^

In this work, a CRISPR-Cas9 lentiviral sgRNA library was infected into a telomerase-immortalized human cell line that expressed Flag-tagged Cas9 and a total of 27 genotoxic agents were used for screenings. From gene level depletion scores, genes were identified, whose mutation/deletion led to a selective growth advantage of the exposed cells. A total of 864 genes were identified whose loss resulted in drug sensitization and 58 genes whose inactivation resulted in drug resistance. The genes covered nearly all DNA repair pathways, DNA replication, regulation of chromosome organization and covalent chromatin modification. About 45% of them belonged to DNA repair groups, indicating that most of the identified genes encode new DDR players, associated with pathways that indirectly influence the response to genotoxic agents. A set of 197 DNA damage repair and signaling factors was grouped according to their function and assigned to the DNA repair sensitization profile of genotoxins, leading to new insights into agent-specific repair and defense programs.

An example was provided by pyridostatin, which binds to G-quadruplex sequences. Pyridostatin clustered with etoposide and doxorubicin, well-known topoisomerase II (TOPO2) inhibitors, and *TDP2, NBS1,* and *ZATT* were hits linked to hypersensitivity of exposed cells. The encoded proteins were previously shown to be involved in the removal of DNA-bound TOPO2, indicating that pyridostatin-induced cytotoxicity is due to poisoning of TOPO2. Another agent, CX5461, previously shown to be a G4-ligand, appeared to act similar to pyridostatin, and inactivation of TDP2 sensitized cells to CX5461, indicating that trapping of TOPO2 is involved. CX5461 shows antitumor activity and thus, it will be interesting to see whether it is a therapeutic alternative to doxorubicin, which causes severe side effects such as cardiotoxicity.

Searching for new players in the canonical DNA repair pathways (Fig. 1), gene activity profiling was performed and revealed that the primase/DNA polymerase *PRIMOL* contributes to benzo[a]pyrene diol epoxide resistance, *ATAD5 (ELG1)* to alkylating agent resistance, and *DSCC1* and *CHTF8* to resistance against a wide variety of genotoxins. ATAD5 is a component of the replication factor C (RFC) complex, which is required for removal of DNA-bound PCNA, while DSCC1 and CHTF8 are components of an alternative RFC complex. Network analysis revealed that the HIPPO pathway, with loss of NF2 and others, sensitized cells to multiple DNA-damaging agents. The pathway is likely to interact with KIRREL and FAM49M—all of them were modulators of chemosensitivity, including genotoxic anticancer therapeutics.^3^

The DNA damage network revealed by CRISPR-Cas9 screenings was also useful to brighten the function of understudied DNA repair factors such as ERCC6L2, which was put into context with a subcluster containing the core NHEJ proteins XRCC4, Lig4, NHEJ1, and the 53BP1-pathway modulators 53BP1, RIF1, and SHLD1-3, as well as the regulator MRI (CYREN). The study revealed that the ATPase ERCC6L2 is part of this subcluster. It is highly interesting that *ERCC6L2* mutations are causally associated with the inherited bone marrow failure syndrome, myelodysplastic syndrome as well as other, not well-characterized clinical phenotypes such as microcephaly and ataxia. *ERCC6L2* mutations cause genomic instability and thus, it was hypothesized that ERCC6L2 is a hitherto unrecognized NHEJ factor (Fig. 1), a supposition that was elegantly verified by knockdowns that turned out to be hypersensitive to etoposide and bleomycin. Overall, the data revealed that ERCC6L2 is a novel NHEJ factor and the root cause of bone marrow failure and other syndromes, in which DSBs are not repaired appropriately.

Having a glance at the nucleotide excision repair (NER) gene cluster, ELOF1, a CSB/RNApol2 associated protein, was identified, whose loss resulted in hypersensitivity to UVC-light, illudin S, and cisplatin. Interestingly, its loss led, at the same time, to resistance to trabectedin, indicating that TC-NER factors are required for eliciting trabectedin cytotoxicity. A similar case exists for *O*^*6*^-alkylating agents such as MNNG and the anticancer drug temozolomide, for which inactivating mutations of mismatch repair genes lead to cellular resistance.^4^ ELOF1 appears to represent an evolutionary conserved modulator of transcriptional inhibition upon RNA polymerase-blocking lesions. Another CSB interacting protein involved in transcriptional recovery following UVC-light is STK19. Its loss led to illudin S sensitization and trabectedin resistance, indicating it belongs, like ELOF1, to the transcription-coupled NER pathway.

Genes involved in the repair of DNA interstrand-crosslinks (ICL) belong to the Fanconi anemia/ICL cluster. A recently discovered ICL factor is HROB.^5^ Other previously uncharacterized genes in this cluster are *CCRR1* and *TXNDC17*. *CCAR1* is associated with DNA damage-induced apoptosis, while inactivation of *TXNDC17* sensitized cells to methylating genotoxins and cisplatin, but not KBrO_3_. From this, and other data, the authors concluded that TXNDC17 is an oxidoreductase that modulates the redox state of thiols (other than GSH), having impact on alkylating agent sensitivity. Another oxidoreductase is CYB5R4 whose inactivation causes diabetes in mice treated with the methylating drug streptozotocin. The protein also seems to be involved in modulating PP2A-family phosphatases, which are required for dephosphorylating γ-H2AX, which is a hallmark of DDR.

The new insights obtained by means of CRISPR-Cas9 genotoxicity screening demonstrate impressively the impact of the method. A limitation is the use of a single cell line, which was mutated for p53. Since p53 regulates several DNA repair genes such as DDB2 and XPC and is involved in cell cycle, cell death, senescence and autophagy regulation, it can be expected that similar studies with other cell systems and on a p53 wild-type background will provide further insights into the complex scenario of DNA repair, DDR and downstream players regulating the fine-tuned balance between survival and death.
